# Supplementary material for: Identification of photoperiod-induced specific miRNAs in the adrenal glands of Sunite sheep (Ovis aries)
Source: Front Vet Sci. 2022 Jul 22;9:888207. doi: 10.3389/fvets.2022.888207 (PMC9354845; doi:10.3389/fvets.2022.888207)
Supplement: Supplementary file 1 [file Data_Sheet_1.ZIP › Supplementary file/miRDeep2 file.zip!/miRDeep2 file/Know_Structure/LP42A3/oar-mir-106a.pdf]

miRBase precursor : oar-mir-106a
Total read count : 75
oar-miR-106a read count : 74
remaining reads : 1

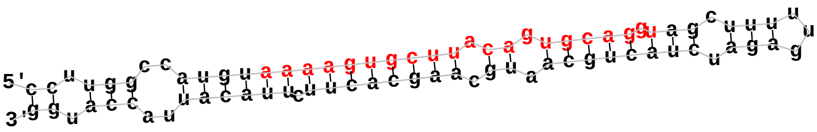

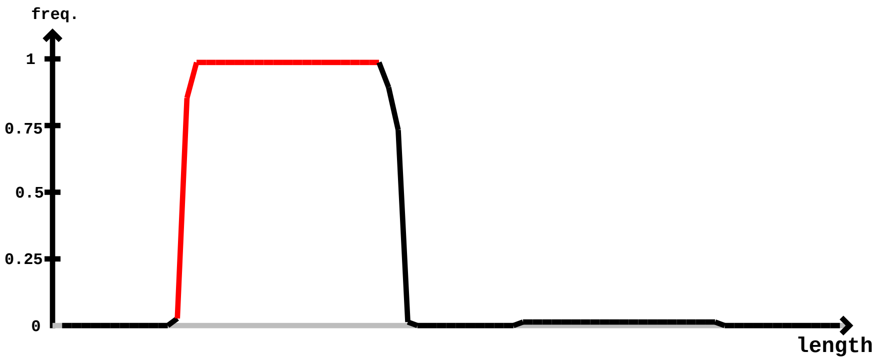


```
                oar-miR-106a
5'- ccuuggccauguaaaagugcuuacagugcagguagcuuuuugagaucuacugcaaugcaagcacuucuuacauuaccaugg   -3'    exp
    ((.(((..((((((((((((((.((.(((((.(((.(((...))).)))))))).)).)))))))).))))))..))).))    reads      mm        sample
    ...........uaaaagugcuuacagugcaggu................................................    1          0         seq
    ...........uaaaagugcuuacagugcagguag..............................................    1          0         seq
    ............aaaagugcuuacagugcaggu................................................    4          0         seq
    ............aaaagugcuuacagugcaggua...............................................    11         0         seq
    ............aaaagugcuuacagugcagguag..............................................    46         0         seq
    ............aaaagugcuuacagugcagguagc.............................................    1          0         seq
    .............aaagugcuCacagugcaggu................................................    1          1         seq
    .............aaagugcuuacagugcaggu................................................    1          0         seq
    .............aaagugcuuacagugcaggua...............................................    1          0         seq
    .............aaagugcuuaUagugcagguag..............................................    1          1         seq
    .............aaagugcuuacagugcagguag..............................................    6          0         seq
    ...............................................uacugcaaugcaagcacuucu.............    1          0         seq
```
